# *In vitro* detection of adrenocorticotropic hormone levels by fluorescence correlation spectroscopy immunoassay for mathematical modeling of glucocorticoid-mediated feedback mechanisms

**DOI:** 10.1186/1687-4153-2012-17

**Published:** 2012-10-26

**Authors:** Martin Gerald Puchinger, Clemens Alexander Zarzer, Philipp Kügler, Erwin Gaubitzer, Gottfried Köhler

**Affiliations:** 1 Department of Structural and Computational Biology, Max F. Perutz Laboratories (MFPL), University of Vienna, Campus-Vienna-Biocenter 5, Vienna, 1030, Austria; 2 Johann Radon Institute for Computational and Applied Mathematics (RICAM), Austrian Academy of Sciences, Altenbergerstr. 69, Linz, 4040, Austria

**Keywords:** ACTH, FCS, AtT-20, Cortisol, CRH, Glucocorticoid membrane receptor, ODE model, Parameter identification

## Abstract

Performing quantitative, highly sensitive measurements at a single molecule level is often necessary to address specific issues related to complex molecular and biochemical systems. For that purpose, we present a technique exploiting both the flexibility of immunoassays as well as the low operating costs and high throughput rates of the fluorescence correlation spectroscopy (FCS) method. That way we have established a quantitative measurement technique providing accurate and flexibly time resolved data of single molecules. Nanomolar changes in adrenocorticotropic hormone (ACTH) levels have been detected in a short time-frame that are caused by fast feedback actions in AtT-20 anterior pituitary glands *in vitro*. Especially with respect to clinical diagnostic or mathematical modeling this improved FCS setup may be of high relevance in order to accurately quantify the amounts of peptide hormones—such as ACTH—as well as signaling molecules, transcription factors, etc., being involved in intra- and extracellular reaction networks.

## Introduction

**Figure 1:**
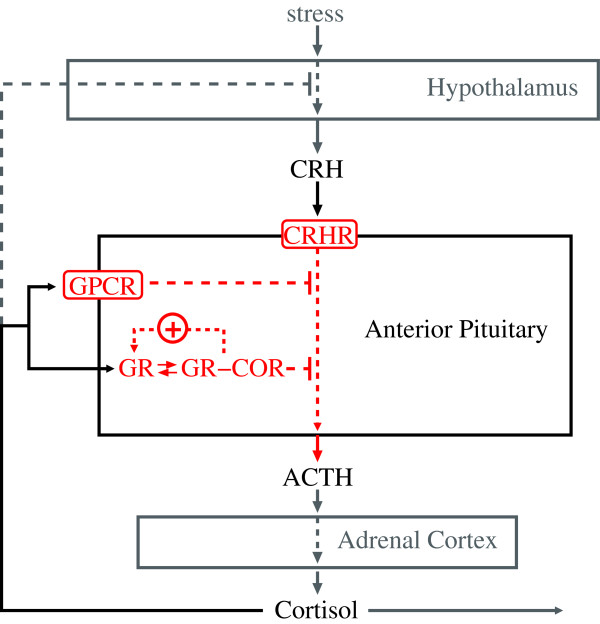
**Schematic representation of the key mechanisms in HPA-axis glucocorticoid-mediated feedback actions.** Two membrane receptors (GPCR, CRHR) mediate extracellular concentrations of cortisol and CRH, inhibiting and stimulating the secretion of ACTH, respectively. In response to a variety of external stressors, CRH is released from the hypothalamus and stimulates the anterior pituitary via CRH-receptors (CRHR) to immediately secrete ACTH, which in turn stimulates the adrenal cortex to synthesize and release cortisol. Thereby non-genomic signaling mechanisms mediate tethering and fusion of ACTH vesicles to the plasma membrane of corticotrophic cells and the fast secretion of ACTH molecules into the extracellular space within minutes after CRH administration. Whereas G-protein-coupled receptors (GPCR) are thought to mediate fast negative feedback actions of glucocorticoids (e.g., cortisol) which downregulate (CRH-induced) ACTH secretion in the anterior pituitary gland. The cytoplasmic organelles such as endoplasmic reticulum, Golgi apparatus, or vesicles are neglected for simplicity.

Adrenocorticotropic hormone (ACTH) is a 39-amino acid long straight-chain peptide hormone (4.5 kDa) that is derived from a 266-amino acid precursor pro-opiomelanocortin. It is secreted by the anterior pituitary gland and is considered one of the major stress hormones within the hypothalamic–pituitary–adrenal (HPA)-axis system: The hypothalamus secrets corticotrophin-releasing hormone (CRH), which stimulates the release of ACTH in the corticotrophic anterior pituitary gland [1]. Consequently, ACTH causes the production of cortisol in the adrenal glands. However, beside corticotrophic feedback actions several other feedback controls on the metabolomic or genomic level provide a complex and multifaceted system. One of the most prominent and well-studied feedback controls is the down-regulation of ACTH production by cortisol. The down-regulation is mediated via two feedback mechanisms working on a genomic and non-genomic levels (see Figure 1). Hence, we observe fast (within seconds to minutes) and slow (after several hours) negative feedback actions in response to the exposure with cortisol [2]. These feedback mechanisms are still subject of research and particularly their interplay is not fully understood. Hence, as ACTH represents the main response in regard to this glucocorticoid feedback, an accurate detection of *in vitro* extracellular ACTH concentration is of high relevance.

The fluorescence correlation spectroscopy (FCS) has proven to be a powerful tool for studying supramolecular associations [3,4], DNA hybridization reactions [5], and detecting single molecule concentrations [6,7]. Due to its high sensitivity, short analysis time and small sample volume requirements FCS have become a valuable tool in molecular biology.

In this article, we present an improved FCS setup to detect nanomolar changes of peptides *in vitro* by combining the fast FCS technique [8] with the highly specific routines of an immunoassay. We exemplify this procedure by means of the *in vitro* measurement of the ACTH peptide secretion from AtT-20 mouse pituitary cells. Particularly, we use a labeled monoclonal antiACTH antibody (specific for the *N*-terminal epitope on the ACTH peptide) to capture the ACTH molecule, making it visible for the FCS. However, in order to detect low molecular weight peptides such as ACTH, the binding of a second unlabeled monoclonal antiACTH antibody to the C-terminal site of the ACTH peptide is necessary in order to cause a significant change in the diffusion time between the free labeled antibody and the mAb(*N*)-ACTH-mAb(*C*) immunocomplex. By measuring this discrepancy in the FCS, the concentration of the target peptide can accurately be determined.

## Materials and methods

### Cell culture

The used AtT-20 cells (ATCC no. CCL-89) were purchased from the American Type Culture Collection (ATCC, Manassas, USA) and passaged at a subcultivation ratio of 1:4 every 5 days. Cells were seeded onto polystyrene 24-well tissue culture plates (Nalge Nunc International, Japan) at a density of 1.0 × 10^4^ cells/ml, grown in Dulbecco’s Modified Eagle’s Medium (Sigma-Aldrich Inc., St. Louis, USA) supplemented with 10% fetal bovine serum, 1.5 g/l sodium bicarbonate, 10 Units/ml penicillin, and 10 μg/ml streptomycin, and maintained in an incubator (HERAcell**®**, Thermo Scientific, USA) at 37°C, 6% CO_2_ and 95% relative humidity. After 92 and 114 h of cell growing, AtT-20 cells were exposed to doses of 10 nM CRH and up to 100 nM cortisol (both from Sigma-Aldrich Inc.) for 1 min to 1 h. The supernatant was carefully removed from the cell layer and centrifuged (800 × g, 37°C, 10 min).

### FCS

**Figure 2:**
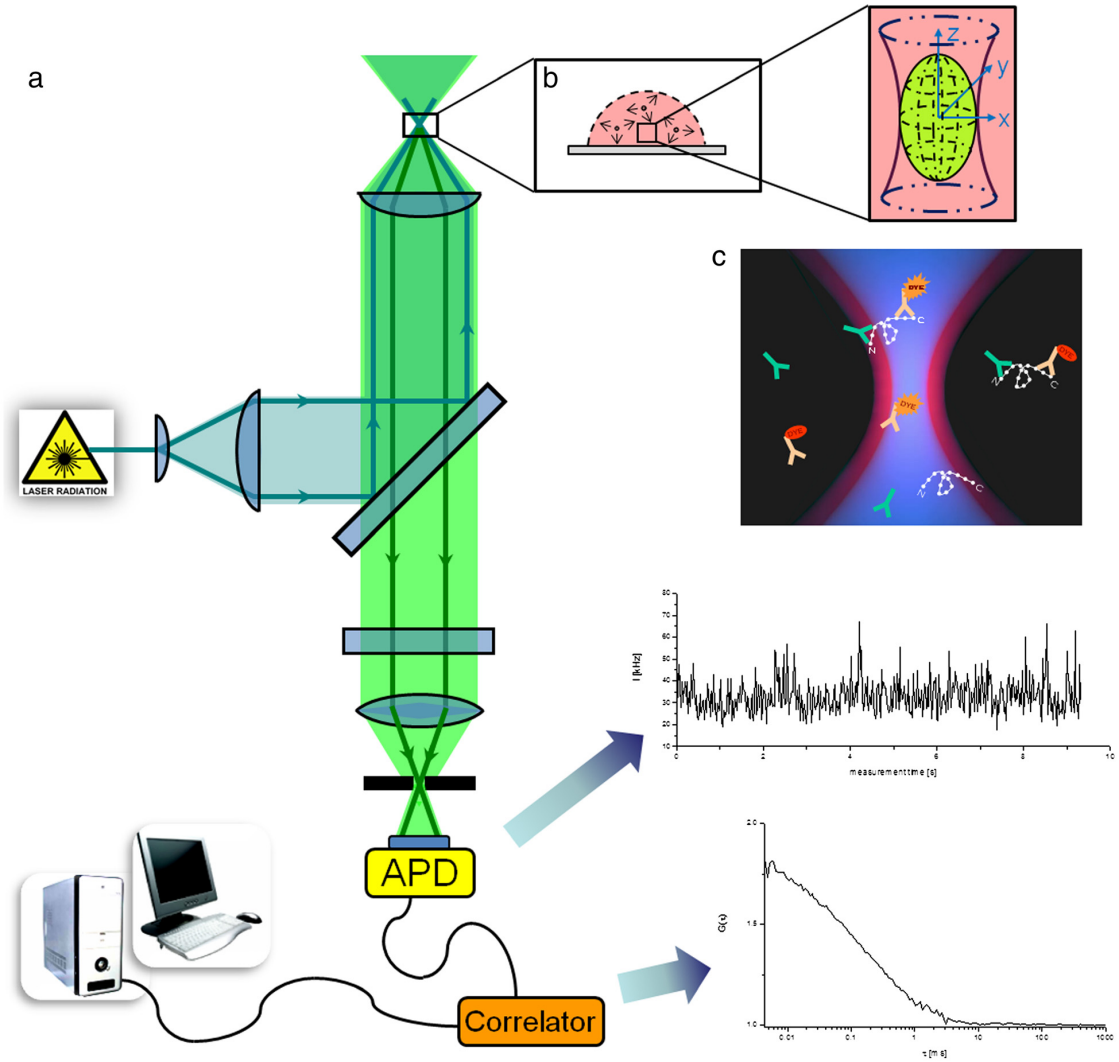
**FCS principle and instrumentation.** In the confocal setup **(a)** of a single-color FCS, the excitation Argon-laser light is directed by a dichroic mirror into a water immersion objective that focuses the light in a calibrated volume inside the sample **(a)**. Changes in diffusion behavior of fluorescent molecules entering and leaving the detection volume are monitored **(a)**. Thereby, each fluorescence signal is collected through the same objective and focused onto a pinhole, so that the laser beam waist inside the sample is imaged onto the pinhole aperture. The conjugation of the objective and the pinhole creates a spatial filter, which efficiently cuts the sampling volume to a diffraction limited size. After the pinhole, the fluorescence signal is collected directly by an Avalanche photodiode and processed into an autocorrelation function *G*(τ) to calculate single molecule concentrations.

FCS measurements were performed on a Confocor spectrofluorimeter (Carl Zeiss-Evotec, Jena, Germany) equipped with an air-cooled 488 nm Argon-laser (LASOS Lasertechnik GmbH, Jena, Germany) and a water immersion objective (C-Apochromat 63 × /1.2 W Korr). The intensity of the laser was set to 70 μW. Intensity fluctuations were recorded by an avalanche photodiode (SPCM-CD 3017) in photon counting mode, autocorrelated with a hardware correlator (ALV 5000, ALV, Langen, Germany), and analyzed with the *FCS ACCESS* (Carl Zeiss-Evotec) software package using a multicomponent fit model (see Figure 2).

#### Focus control and pinhole adjustment

A drop of the organic fluorescent dye Rhodamine 6 G (diluted 1:200) was used to automatically position the chambered coverglass (Nalge Nunc International, Japan) in focus of the confocal optics of the spectrofluorimeter by a scanning procedure as well as to automatically adjust the pinhole to its correct position. The focus in *z*-direction was set 150 μm over the coverglass to record diffusions of fluorescent particles through the focal element in the drop of sample. The pinhole diameter was set to 35 μm.

#### Calibration of confocal volume

The confocal detection volume was determined by measuring the correlation time of a 10-nM solution of rhodamine 6 G in water with the known diffusion coefficient *D* of 2.8 × 10^–6^ cm^2^/s, employing the relationship *D* = *r*^2^/(4τ_*D*_) and resulting in a confocal volume element of 0.17 μm in radian and 0.88 μm in axial dimension. In addition, DyLight488-labeled monoclonal antibody dilution series with known concentrations (ranging from 1 to 80 nM) were measured and showed similar molarities in FCS. Confocal volume calibrations using rhodamine 6 G were carried out on each experimental day or after 3 h measurement time.

#### FCS immunoassay

60 nM antiACTH(*N*-term)-monoclonal IgG1 antibodies (Phoenix Pharmaceuticals Inc., Belmont, USA) and 60 nM-labeled antiACTH(*C*-term)-IgG1 antibodies (Fitzgerald Industries International, Concord, USA; labeled with DyLight488 from Pierce Biotechnology, Rockford, USA) were added in the cell-free supernatant and incubated (30°C, 15 min) to ensure a quick and absolute ACTH-capture of both monoclonal antibodies. Western blotting analysis and ELISA measurements prior to FCS showed no crossreactivity between both antibodies in absence of ACTH. A drop (25 μl) of sample was pipetted on the chambered coverglass, excited with an 488-nm Ar-laser attenuated by an optical density filter (1.0–in. diameter), and the fluctuations in fluorescence intensity of the mAb(*N*)-ACTH-mAb(*C*) immunocomplex compared to the free labeled antibody were monitored in series of 50 measurements with identical setup (measurement time: 10 s; correlator scaling: 10 s) for each sample (see Figure 2).

### Calculation

Statistical analysis of the autocorrelation function by a 2-component fitting procedure computationally distinguishes the labeled unbound antibody fraction from the desired ACTH-bound form (immunocomplex). The normalized autocorrelation function *G*(τ) describes the fluctuations of a signal *F*(*t*) from the mean intensity at any time compared to fluctuations at any later time *F*(*t* + *τ*). It is given by

$$G\left(\tau \right)=\frac{〈\delta F\left(t\right),\;\delta F\left(t+\tau \right)〉}{{〈F\left(t\right)〉}^{2}}\text{,}$$

where the angular brackets in the function represent the ensemble average, δ*F*(*t*) denotes the corresponding variance, and τ is known as the delay or correlation time interval over which the fluctuations are compared.

For a single diffusing species (a one-component model) in a Gaussian confocal volume, the autocorrelation function *G*(τ) is defined by [3]

$$G\left(\tau \right)=1+\frac{1}{N\left(1+\frac{\tau }{\tau_{D}}\right)\sqrt{1+\frac{\tau }{{\left(\frac{z}{r}\right)}^{2}\tau_{D}}}}\text{,}$$

where *N* is the particle number and τ_*D*_ the molecular diffusion time of the excited fluorophores moving in a three-dimensional confocal volume through an axial (*z*) to radial (*r*) dimension.

The molecular diffusion time for a one photon excitation is given by the following relationship to the diffusion coefficient *D* [cm^2^/s]

$$\tau_{D}=\frac{r^{2}}{4D}$$

The obtained autocorrelation functions were evaluated using a two-component model by fixing the diffusion time of the unbound labeled antibody fraction (τ_*D*1_) which was achieved from one-component fitting procedure.

The analytical formula for the two-component model, which was successfully applied in a previous work [3], was used in a modified form and is given by

$$\begin{array}{l} G(\tau )=1+\frac{1}{N^{\prime }}\left[\left(1-Y\right)g_{D1}\left(\tau \right)+Yg_{D2}\left(\tau \right)\right]\\ with\;N^{\prime }=N_{1}+N_{2}\\ g(\tau )={\left(1+\frac{\tau }{\tau_{D}}\right)}^{-1}{\left(1+\frac{\tau }{{\left(\frac{z}{r}\right)}^{2}\tau_{D}^{2}}\right)}^{-0.5} \end{array}$$

**Figure 3:**
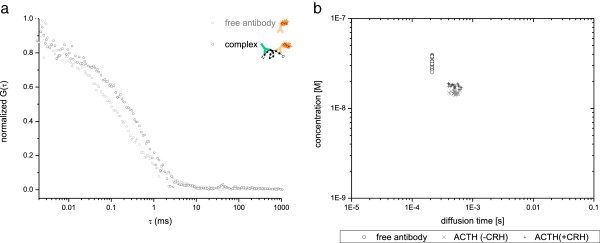
**FCS studies of a single and two diffusion species.** (**a**) The two autocorrelation functions were calculated from intensity fluctuations of the free antibody and that of the ACTH-bound ones. The shift of the autocorrelation curve (**a**, blue line) to the right indicates that the diffusion time of the ACTH-bound particles through the focus is higher than that of the free antibody. To eliminate the amount of unbound from the ACTH-bound forms in the sample, a two-component fit over the autocorrelated data points of the ACTH-bound form (**a**, blue line) was performed with a fixed diffusion time for the unbound antibodies (220 μs, previously calculated with a one-component fit to obtain the average diffusion time of free unbound fluorescent antibodies) and a variable one for quantifying different ACTH concentrations. (**b**) A double-logarithmic plot of calculated concentrations versus related diffusion times over 50 measurements shows the accuracy of the results obtained by FCS for different ACTH concentrations.

This yields values of diffusion times (τ_*D*1_, τ_*D*2_) and of the related mole fractions *Y* and (1 – *Y*) for the two components. Autocorrelation analysis was performed for a fixed structural parameter of 5 defining the ratio between the height and the width of the detection volume. This parameter was obtained from calibration with rhodamine 6 G in water. The fit model determines the average number of fluorescent molecules within the detection volume, and the characteristic diffusion times. Evaluations of the autocorrelation function of only labeled antibodies result in diffusion times of τ_1_ = 220 ± 8 μs (mean ± *t*-student) through the confocal volume using a one-component fitting procedure. These results are in accordance to the diffusion time of the IgG-antibody (*D* of 3.7 ± 0.2 × 10^–7^ cm^2^/s) of 200 μs calculated by Stokes–Einstein relation. Formation of the immunocomplex results in a characteristic diffusion behavior of τ_2_ = 483 ± 83 μs (mean ± *t*) through the confocal volume compared to freely labeled anti-ACTH IgG antibody in solution (τ_1_ fixed to τ_1_ = 220 μs; see Figure 3).

As the amplitude of an autocorrelation function is inversely proportional to the average number of fluorescent particles within the confocal volume (*V*_conf_ of 5 × 10^–16^ l)

$$G\left(0\right)=\frac{1}{N_{D1,2}}\text{,}$$

the absolute concentration of free labeled IgG antibodies (*N*_*D*1_) and of the ACTH immune complexes (equal to number of ACTH molecules; *N*_*D*2_ = *N*_ACTH_) can be obtained by

$$\begin{array}{l} c_{\text{ACTH}}=\frac{N_{\text{ACTH}}}{6.023\cdot 10^{23}{\text{mol}}^{-1}\cdot 5\times 10^{-16}\;1}\\ \;=\frac{N_{\text{ACTH}}}{30.115\times 10^{7}}\text{mol}/1 \end{array}$$

$$\;c_{\text{IgG}}=\frac{N_{\text{IgG}}}{30.115\times 10^{7}}\text{mol}/1$$

## Results

### Dose response relation of CRH and cortisol to ACTH secretion

**Figure 4:**
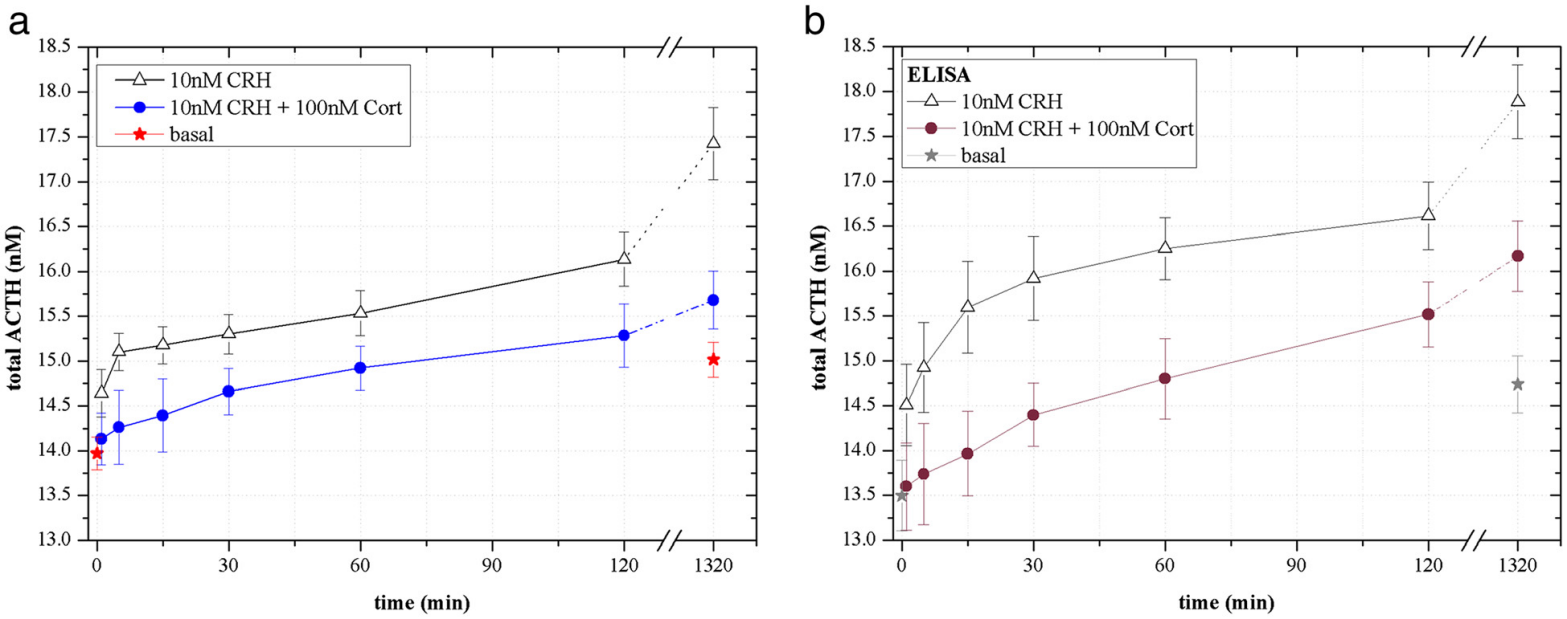
**Fast and delayed negative feedback regulation of ACTH release *in vitro *.** A typical HPA response to a strong extracellular stress signal (10-nM CRH) is simulated in AtT-20 pituitaries *in vitro * by adding 100-nM cortisol. Already minutes after cortisol incubation an inhibition and a delay of extracellular ACTH secretion is detectable (blue circular data points), which is supposed to be caused by a fast negative feedback mechanism. Long-term inhibitory effects of cortisol to ACTH secretion (interrupted line) were not analyzed. FCS datasets (**a**) are consistent with ELISA control measurements (**b**). Deviations calculated by the *t*-test distribution, α = 0.1.

The experimental results show a basal ACTH secretion which is not affected by extracellular CRH and cortisol signals (see Figure 4) and thus it seems not to be modulated by the main feedback controls. Moreover, our data feature a strong ACTH response (17.427 ± 0.422 nM) to an extracellular dose of 10 nM CRH over 22 h (compared to that of 5 and 36 nM), indicating an increased ACTH secretion in the AtT-20 cells *in vitro*. In addition to an extracellular dose of 10-nM CRH, an administration of 50 nM cortisol partly inhibited the stimulated ACTH secretion after 22 h compared to that in absence of cortisol. A maximal inhibition of the ACTH release was achieved by adding 100-nM cortisol leading to ACTH levels close to the basal one (Table 1).

**Table 1:** ***In vitro *studies in AtT-20s within 22 h**

**CRH (nM)**	**Cort (nM)**	**Cell population (cells/ml)**	**ACTH (nM)**
0	0	205164 ± 12889	15.016 ± 0.201
5	0	335938 ± 20522	16.764 ± 0.593
**10**	**0**	324219 ± 23935	**17.427** ± **0.422**
36	0	302084 ± 28168	16.146 ± 0.564
54	0	237500 ± 15630	15.364 ± 0.575
10	50	312500 ± 28125	16.673 ± 0.467
**10**	**100**	298438 ± 20625	**15.679** ± **0.337**

Cell proliferation and ACTH response to various doses of extracellular CRH and cortisol within/over 22 h of incubation (114 h total incubation time). Optimal concentrations for CRH stimulation and cortisol inhibition of ACTH secretion were used for studying feedback regulation of ACTH release (bold values). Deviations calculated by the *t*-test distribution, α = 0.1.

### Fast feedback regulation of ACTH release

In order to demonstrate the capabilities of the method we focused on the fast negative feedback control by cortisol. Due to the fast sampling and the low sample volume we were able to detect significant differences in ACTH response within 5–15 min after CRH and/or cortisol incubation (see Figure 4).

### Validation of FCS results

FCS and immunoassays (with chemiluminescent, fluorescent, or HRPO signals) are both known as quite sensitive detection techniques. Thus, a two-site ELISA (MDBioscience, Switzerland) is used to validate the data obtained by FCS. Table 2 and Figure 4 show that the ELISA results are consistent with the detected FCS datasets.

**Table 2:** Validation of FCS results

**Time (min)**	**ACTH (FCS)**		**ACTH (ELISA)**	
	**% change**^**a**^	**% dev**	**% change to FCS**	**% dev**
Basal				
0	0.00	±1.28	−3.39	±2.91
1320	+7.48	± 1.29	−1.86	±2.16
+10 nM CRH				
1	+4.79	±1.82	−0.91	±3.11
5	+8.08	±1.39	−1.16	±3.35
15	+8.62	±1.36	+2.77	±3.29
30	+9.51	±1.43	+4.05	±2.96
60	+11.18	±1.62	+4.61	±2.12
120	+15.50	±1.86	+2.97	±2.27
1320	+24.74	±2.32	+2.63	±2.31
+10 nM CRH + 100 nM cortisol				
1	+1.16	±2.03	−3.77	±3.58
5	+2.08	±2.88	−3.68	±4.12
15	+3.02	±2.85	−2.97	±3.38
30	+4.94	±1.75	−1.80	±2.44
60	+6.80	±1.63	−0.81	±3.01
120	+9.40	±2.31	+1.52	±2.33
1320	+12.23	±2.07	+3.10	±2.41

Validation of FCS results by ELISA measurements. The % difference in ACTH concentrations obtained from ELISA measurements refers to the FCS dataset of the same sample (in the same row in the table).

^a^The temporal % change of ACTH concentration to the basal ACTH-level of 13.9709 nM (mean value) detected by FCS. Deviations calculated by the *t*-test distribution, α = 0.1.

### Mathematical modeling of feedback mechanisms

The improved setup for the FCS method is particularly suitable for experiments which have to be conducted repeatedly and demand a high (quantitative) accuracy of the data. A central motivation to develop such a technique comes from a mathematical modeling task initiated by the research presented in [9]. We are interested in the interplay of the genomic and non-genomic negative feedback of cortisol on the secretion of ACTH and its effect on the dynamics of the HPA-axis. This research goal demands to model both intracellular mechanisms as well as interactions of the different glands. This cannot be achieved in full detail. Consequently, we concentrated on the main feedback mechanisms related to the anterior pituitary gland and the basic controls between the hypothalamus and the adrenal glands. This approach of bridging several levels of complexity eventually needs validation by experimental data. In that regard the measurement of the ACTH secretion in response to CRH and cortisol serves two goals. First of all the secretion of ACTH is targeted by the main feedback controls we focus on in our model and thus allows us at least a basic assessment of the model. And secondly, we introduce no bias concerning the overall dynamics of the HPA-axis, as we consider only the anterior pituitary cells in our *in vitro* experiment and thus have no interaction with other tissues or glands.

**Figure 5:**
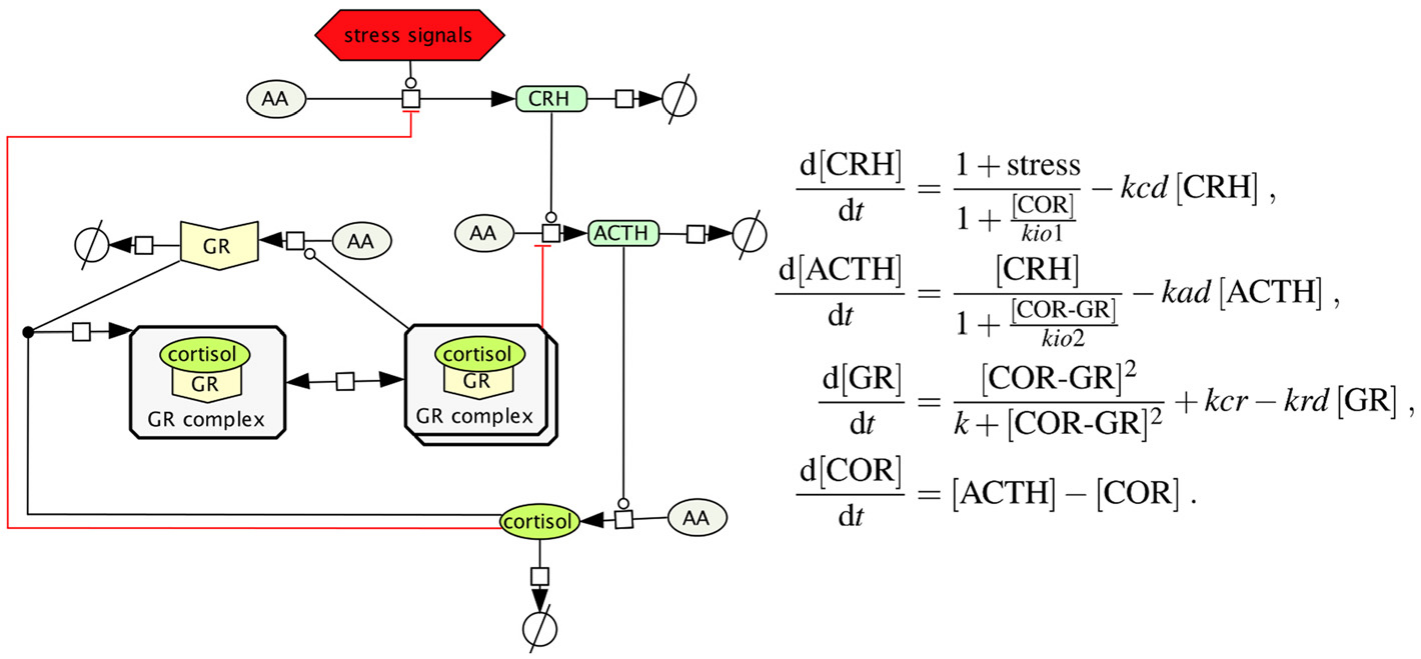
**Parsimonious model of the HPA-axis.** The figure shows a graphical outline and the corresponding mathematical equations of the parsimonious model as published in [10]. The focus of this model is the negative feedback of cortisol on the production of ACTH via an intracellular glucocorticoid receptor, which acts as a transcription factor in its dimerized form. Eventually, the model offers an intriguing explanation for the widely observed scenario of hypocortisolism.

**Figure 6:**
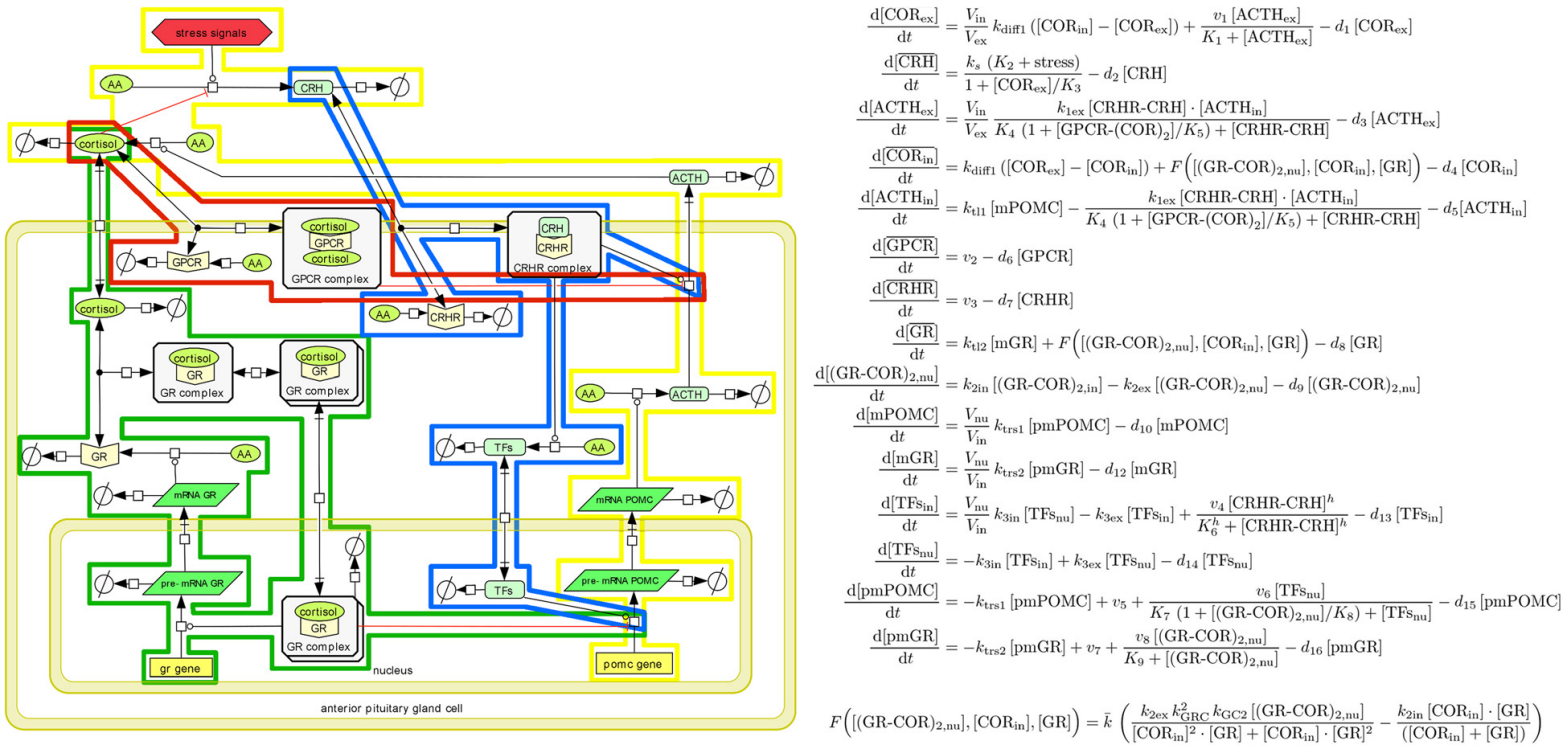
**Mathematical model of the HPA-axis focussing the anterior pituitary gland.** Outline of an extended mathematical model following the lines of the modeling approach in [10]. The image on the left-hand side provides an overview of the considered molecular mechanisms and feedback controls. In particular, it shows the three different modeled compartments, i.e., the extracellular space, the intracellular space of the pituitary gland cells, and their nucleus compartment. On the right-hand side, the equations of the ODE system are given. Based on the mathematical model from [10], these equations are deduced by introducing the different compartments and in particular two membrane receptors of the pituitary gland cells.

With respect to the modeling technique we followed an approach in [10] which also focused on the anterior pituitary cells. Figure 5 provides a sketch of the considered feedback controls and the used mathematical equations in [10]. In Figure 6, we provide a graphical outline of our model and the corresponding mathematical description as set of the ordinary differential equations. Our extended model follows the approach offered in [10] but in addition considers the fast non-genomic feedback mechanism via the glucocorticoid membrane receptor in the anterior pituitary cells (red-framed pathway). Moreover, the model includes the slow genomic feedback mechanism of cortisol (green-framed pathway) and CRH-mediated genomic and non-genomic effects (blue-framed pathway). To the best of the authors’ knowledge the proposed model is the first one of the HPA-axis which incorporates central receptors. Consequently, it differs significantly in size and structure from most models present in literature, which focus on most prominent species cortisol, ACTH, CRH, and vasopressin (cf. [11-14]). Moreover, our model is novel in the sense that we explicitly model an intracellular compartment and the nucleus. This allows us to take translocation processes into account and thus differ between genomic and non-genomic effects.

**Figure 7:**
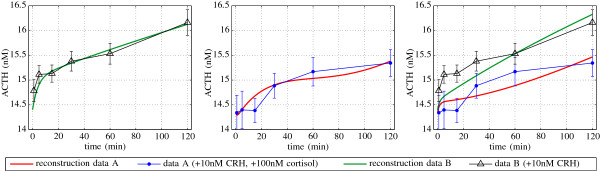
**Fit of the experimental data by means of the parsimonious model from**[10]**.** The figure shows the result of a numerical experiment for the obtained experimental data and the mathematical model as published in [10]. The first (left) plot shows the result of a parameter fit for the dataset without added cortisol. The second (middle) plot shows the reproduction of the ACTH response curve in the presence of CRH and cortisol. The last (right) plot presents the result when considering both experimental datasets at once. The poor quality of the obtained fit in the latter case indicates that the parsimonious model is not capable to reproduce the fast and slow dynamics related to the negative feedback via cortisol.

In a first attempt we used the model from [10] and tried to reproduce the data obtained by means of our FCS method. Figure 7 shows that if we consider only one of the conducted experiments, i.e., only a dosage of CRH or the scenario of adding CRH as well as cortisol, the model is capable to reproduce the basic dynamics. However, we were not able to find any parameter set such that the parsimonious model is able to exhibit both scenarios. The parameter space was searched using the genetic algorithms from the MATLAB global optimization toolbox. In particular, we used different initial populations of about 10^4^ to 10^5^ elements. The objective functional was based on Tikhonov-type regularization functional (c.f. [9] for instance).

**Figure 8:**
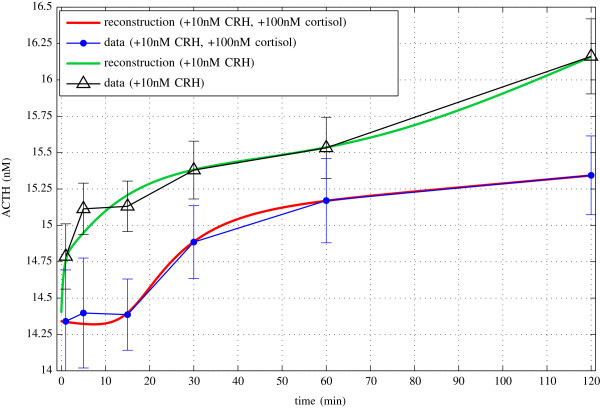
**Fit of the experimental data by means of the multicompartment model.** The graph shows the result of the parameter fitting for the extended mathematical model, including the membrane receptors for cortisol and CRH. The identified parameters allow to reproduce the observed dynamics in the two scenarios, i.e., with and without cortisol. Hence, by using different initial values for the ODE state variable both characteristic dynamics can be simulated with the same set of parameters. In particular, in the presence of cortisol the fast negative feedback can be reproduced, leading to the experimentally observed sigmoidal curve.

Figure 8 shows the data fit computed for our extended model. The parameter fit was computed by the Tikhonov regularization, where we used standard global and local optimization algorithms to minimize the objective functional. The extended model allows to correctly reproduce the observed ACTH response behavior. The fact that the model is capable to reproduce the experimental data indicates at least a feasible model and, particularly, supports the underlying idea of distinguishing between the genomic and non-genomic feedback mechanisms. We emphasize that the present dataset is neither sufficient nor suited to identify the ‘true’ values of all model parameters. The conducted numerical experiment mainly serves to assess the overall behavior of the extended model, particularly in comparison with the parsimonious model as discussed in [10].

## Conclusion

In 2005, Maier et al. [3] were able to provide evidence of a glucocorticoid receptor in the anterior pituitary cell membrane which may regulate fast response of anterior pituitary cells to cortisol. With our improved FCS setup it was possible to detect lowest changes in extracellular ACTH molarities (±0.3 nM) that arise from signaling of these activated G-protein coupled membrane receptors *in vitro* (see Figure 1). Even 5–15 min after cortisol administration (100 nM) we were able to monitor an inhibition of CRH-induced ACTH secretion by cortisol. Extracellular ACTH levels of 14.387 ± 0.428 nM compared to 15.131 ± 0.254 nM without addition of cortisol were measured. The fast inhibitory effects on CRH-induced ACTH secretion have become evident within at most 5 min after cortisol administration (see Figure 4). However, a detailed temporal restriction of fast and slower feedback actions on extracellular ACTH secretion was not studied with this FCS setup so far, but our results suggested that immediate ACTH secretion which has occurred within minutes after cortisol treatment can only be caused by fast non-genomic feedback actions (see Figure 1) and not by genomic-slow feedback mechanisms which have been shown to occur after several hours [15].

FCS provides a highly flexible, easy-to-use assay format with very small sample volumes (approximately 20 μl), and increased throughput, as particle numbers can be measured directly after calibrating the confocal volume once, a major disadvantage of ELISAs which need to be calibrated quite often. In addition, FCS makes it possible to extract complex signals from high background due to the different characteristic time scales over which signal and noise occur.

In conclusion, we have demonstrated that this improved FCS setup can be used for fast and sensitive detection of a specific peptide hormone *in vitro*. By means of the mentioned model system we established this solution-based single molecule detection technique as an alternative to the commonly used approaches, such as ELISAs (with fluorescent, chemiluminescent, or HRPO signal), with respect to rapidity and sensitivity (Table 3). It was possible to even detect nanomolar changes in ACTH secretion with deviations of only 0.2–0.7 nM approximately in response to an extracellular stress signal over a short period of time. The quality of the data obtained by FCS allowed to study fast feedback mechanisms in the HPA-axis regulatory system *in vitro* and allowed to support the development of a mathematical model of that HPA-axis network. As opposed to [10] our model takes both the genomic and non-genomic feedbacks mechanisms into account. As a result it is able to feature both ACTH response curves with a single set of model parameters.

**Table 3:** FCS-immunoassay versus ELISA

**Parameter**	**FCS-assay**	**ELISA**
Sensitivity	μM to sub-nM	nM to sub-nM
Sample volume required	Approximately 20 μl	100 μl
Measurement time	Approximately 25 min	>2.5 h
Immunocomplex formation	In solution	On the surface
Washing steps required	No	Yes
Calibration curve required	Yes (confocal volume)	Yes
Detection mode	Size-based fluctuations in signal intensities	Changes in signal intensity

## Abbreviations

ACTH: Adrenocorticotropic hormone; CRH: Corticotrophin-releasing hormone; CRHR: CRH-receptor; ELISA: Enzyme-linked immunosorbent assay; FCS: Fluorescence correlation spectroscopy; GPCR: G-protein-coupled receptor; GR: Glucocorticoid receptor; HPA: Hypothalamic–pituitary–adrenal; mAb: Monoclonal antibody; POMC: Pro-opiomelanocortin; TF: Transcription factor.

## Competing interests

The authors declare that they have no competing interests.
